# Isolation and monoculture of functional primary astrocytes from the adult mouse spinal cord

**DOI:** 10.3389/fnins.2024.1367473

**Published:** 2024-02-16

**Authors:** Ingrid L. Peterson, Austin D. Thompson, Natalie E. Scholpa, Tally Largent-Milnes, Rick G. Schnellmann

**Affiliations:** ^1^Department of Pharmacology, College of Medicine, University of Arizona, Tucson, AZ, United States; ^2^Department of Pharmacology and Toxicology, College of Pharmacy, University of Arizona, Tucson, AZ, United States; ^3^Southern Arizona VA Health Care System, Tucson, AZ, United States; ^4^Southwest Environmental Health Science Center, University of Arizona, Tucson, AZ, United States; ^5^Department of Neuroscience, College of Medicine, University of Arizona, Tucson, AZ, United States; ^6^Center for Innovation in Brain Science, University of Arizona, Tucson, AZ, United States

**Keywords:** astrocytes, spinal cord, ACSA-2, mouse, primary, *in vitro*, isolation

## Abstract

Astrocytes are a widely heterogenic cell population that play major roles in central nervous system (CNS) homeostasis and neurotransmission, as well as in various neuropathologies, including spinal cord injury (SCI), traumatic brain injury, and neurodegenerative diseases, such as amyotrophic lateral sclerosis. Spinal cord astrocytes have distinct differences from those in the brain and accurate modeling of disease states is necessary for understanding disease progression and developing therapeutic interventions. Several limitations to modeling spinal cord astrocytes *in vitro* exist, including lack of commercially available adult-derived cells, lack of purchasable astrocytes with different genotypes, as well as time-consuming and costly in-house primary cell isolations that often result in low yield due to small tissue volume. To address these issues, we developed an efficient adult mouse spinal cord astrocyte isolation method that utilizes enzymatic digestion, debris filtration, and multiple ACSA-2 magnetic microbead purification cycles to achieve an astrocyte monoculture purity of ≅93–98%, based on all markers assessed. Importantly, the isolated cells contain active mitochondria and express key astrocyte markers including ACSA-1, ACSA-2, EAAT2, and GFAP. Furthermore, this isolation method can be applied to the spinal cord of male and female mice, mice subjected to SCI, and genetically modified mice. We present a primary adult mouse spinal cord astrocyte isolation protocol focused on purity, viability, and length of isolation that can be applied to a multitude of models and aid in targeted research on spinal-cord related CNS processes and pathologies.

## 1 Introduction

Astrocytes are a morphologically heterogenous cell population that are integral to central nervous system (CNS) homeostasis, including ion, pH, and neurotransmitter regulation (Verkhratsky and Nedergaard, 2018; Verkhratsky et al., 2021). These glial cells also provide structural support, are involved in synaptogenesis and neurogenesis, and are key components of blood-CNS barriers (Verkhratsky et al., 2021). Previously thought to serve primarily as supportive cells, recent research has shown that astrocytes also play an active role in the tripartite synapse, communicating bidirectionally with neurons (Santello et al., 2012).

In addition to their homeostatic functions, astrocytes participate in various neuropathologies, including CNS trauma and neurodegenerative disease progression. The importance of these cells post-injury is widely accepted; however, questions regarding the balance between the positive and negative activities of astrocytes remain (Pekny and Pekna, 2014). For example, following spinal cord injury (SCI), proliferation of reactive astrocytes contributes to the formation of a physical barrier between damaged tissue and spared tissue known as the glial scar (Adams and Gallo, 2018). While this barrier is detrimental to axonal regeneration after injury, the glial scar also restricts the spread of inflammation and fibrotic tissue, which is considered beneficial (Yang et al., 2020). This dichotomy is thought to be dependent on both astrocyte sub-type and time post-injury (D’Ambrosi and Apolloni, 2020; Yang et al., 2020). As such, further understanding of the various roles of astrocytes in spinal cord pathologies has the potential to contribute to the development of new treatment paradigms and time-to-care decisions that could improve quality of life for patients.

*In vitro* studies are often conducted using astrocytes isolated from neonatal mice, despite many neuropathologies occurring more frequently in adults. Importantly, differences in the behavior and morphology of neonatal versus adult astrocytes have been observed in models of SCI (Barrett et al., 1984), ischemic stroke (Revuelta et al., 2019), and traumatic brain injury (TBI) (Dai et al., 2019). Therefore, the ability to use adult astrocytes to more accurately model disease processes could improve the understanding of disease progression and developing therapeutic strategies.

Commonly, isolating primary astrocytes from the adult spinal cord results in a decreased yield due to age and smaller tissue volume compared to the brain. To address this need, we developed a protocol for adult mouse spinal cord astrocyte isolation that results in astrocytes of suitable quality and quantity for downstream *in vitro* experimentation. Described below is the method of isolation from spinal cords of female C57Bl/6 J (naïve) mice, the injury site from female mice that have undergone SCI (SCI mice), female B6.129P2-*Cnr2^tm1Dgen^*/J mice, which lack the spinal cord astrocyte endocannabinoid 2 receptor (CB2R KO), as well as male astrocyte-specific β_2_-adrenergic receptor (ADRB2) knockout (*GFAP-Cre/ADRB2^Flox/Flox^*) mice and littermate controls (*WT-Cre/ADRB2^Flox/Flox^*). Therefore, in addition to isolation, our method allows for long-term culture of adult mouse spinal cord astrocytes from various sources, speaking to the potential for direct correlation between *in vitro* and *in vivo* models.

## 2 Materials and equipment

Concentrations, compositions of solutions, technical notes, and supplier information for all components utilized throughout this protocol can be found in Tables 1–3. Products used for isolation can be found in Table 1, those for characterization in Table 2, and products for functional assessments in Table 3.

**Table 1:** Products used for isolation.

Product	Product number	Manufacturer	Use	Parameters
DMEM/F-12, GlutaMAX^™^ supplement	10–565-018	Gibco	Collection, quench, trituration, cell culture	media+10% FBS and 1% Pen/Strep
Penicillin–Streptomycin (10,000 U/mL)	15–140-163	Gibco	For prevention of bacterial contamination	1% in all applications used
DPBS (+calcium/+magnesium)	14,040,182	Gibco/Thermo Fisher	wash steps after cord collection	2 mL per wash per cord, 6 mL total per cord
Gibco: Fetal Bovine Serum, certified, One Shot^™^ format, United States	A3160401	Thermo Fisher	Nutrition	5% in all applications used
Gibco^™^ HBSS, +calcium/+magnesium, no phenol red	#14025	Gibco	washes after isolation	1% Pen/Strep added; ice-cold 3 mL/cord each wash
DNase I recombinant, RNase-free	4,716,728,001	Sigma/Millipore	degrades DNA	1:10 into buffer then accutase
Accutase® solution	NC1670906 A6964-100ML	Sigma	Tissue digestion	2 mL/cord
Protease inhibitor Cocktail	P8340	Sigma	Accutase neutralization during tissue processing steps	1:100 per manufacturer’s recommendations
Anti-ACSA-2 MicroBead Kit, mouse	130–097-678	Miltenyi Biotec	Tagging and sorting of primary cells	kit FcR Blocker Reagent: 1:50; Anti-ACSA-2 MicroBead: 1:25
Collagen from calf skin	C8919-20ML	Sigma/Millipore	Cell Culture	1:4 in nanopure water, plates treated at 37°C for >2 h prior to platting
Pre-Separation Filters (70 μm)	130–095-823	Miltenyi Biotec	to filter out large pieces of tissue debris and remining myelin	1 filter per cord
MACS® SmartStrainers (70 μm)	130–098-462	Miltenyi Biotec	to filter out clumped cells and any remaining debris	1 filter per sample
FcR Blocking Reagent, mouse	130–092-575	Miltenyi Biotec	blocks unwanted binding of antibodies to Fc receptor-expressing cells	1:50
Phosphate Buffer Saline (PBS; −calcium/−magnesium)	10,010,049	Gibco	General	General
Bovine serum albumin (BSA)	A4503-100G	Sigma-Aldrich	FACS solution	see microbead kit manufacturer’s recommendations
Samco^™^ Transfer Pipettes, 7.7 mL, Sterile Individually Wrapped	202-1S	ThermoScientific	General	General
LS Magnetic Cell Separation Columns	130–042-401	Miltenyi Biotec	Magnetic cell sorting	1 column per sample per wash (3 columns per sample total)
Ibidi USA μ-Dish 35 mm, High, IbiTreat—Tissue Culture Treated Polymer Coverslip	(81156) 50–305-806 Fisher	Fisher	Cell Culture	Treated with collagen prior to plating

**Table 2:** Products used for characterization.

Product	Product number	Manufacturer	Use	Parameters
Anti-GFAP antibody	ab7260	Abcam	ICC	ICC: 1:1000
ACSA-2 Antibody, anti-mouse, PE, REAfinity^™^	130–116-141	Miltenyi Biotec	ICC and Flow Cytometry	Flow: 1:25, ICC: 1:50
GLAST (ACSA-1) Antibody, anti-human/mouse/rat, PE	130–118-344	Miltenyi Biotec	ICC and Flow Cytometry	Flow: 1:25, ICC: 1:50
GFAP Antibody, anti-human/mouse/rat, REAfinity^™^ (APC)	130–123-846	Miltenyi Biotec	Flow Cytometry	Flow: 1:25
recombinant IgG1 monoclonal antibodies, or with recombinant human IgG1 PE+ and APC+ conjugated isotype control antibodies	130–113-438 &130–113-434	Miltenyi Biotec	Flow Cytometry	Flow: 1:25
EAAT2 Polyclonal Antibody	BS-1751R	ThermoFisher	Flow Cytometry	Flow: 1:50
Anti-EAAT2 antibody	ab41621	Abcam	ICC	ICC: 1:500
TMEM119 Rabbit anti-mouse IgG polyclonal antibody	PA5-119902	Invitrogen	ICC and Flow Cytometry	Flow: 1:500, ICC: 1:500
IBA-1 recombinant rabbit anti-mouse monoclonal antibody [EPR16588]	ab178846	Abcam	ICC and Flow Cytometry	Flow: 1:160, ICC: 1:500
Donkey anti-Rabbit IgG (H + L) Highly Cross-Adsorbed Secondary Antibody, Alexa Fluor^™^ Plus 488	A32790	Invitrogen	ICC and Flow Cytometry	Flow: 1:500, ICC: 1:500
Goat anti-Rabbit IgG (H + L) Highly Cross-Adsorbed Secondary Antibody, Alexa Fluor^™^ Plus 555	A32732	Invitrogen	ICC and Flow Cytometry	Flow: 1:1000, ICC: 1:1000
Goat anti-Rabbit IgG (H + L) Cross-Adsorbed Secondary Antibody, Alexa Fluor^™^ 568	A-11011	Invitrogen	Flow Cytometry	Flow: 1:500
Rabbit IgG (H + L) Highly Cross-Adsorbed Secondary Antibody, Alexa Fluor^™^ Plus 647	A32733	Invitrogen	ICC	ICC: 1:1000
Ibidi USA Supplier Diversity Partner Ibidi Mounting Medium With DAPI, 15 mL	NC1943852	Fisher	ICC	7 drops/35 mm plate, 4 drops per 24-well well
Image-IT^™^ Fixative Solutions	I28800	Invitrogen	Cell Fixation	Flow/ICC
Triton^™^ X-100	T8787-100 mL	Sigma-Aldrich	Cell Permeabilization	Flow/ICC
Phosphate Buffer Saline (PBS)	10,010,049	Gibco	General	General

**Table 3:** Products used for functional assessments.

Product	Product number	Manufacturer	Use	Parameters
bEnd.3	CRL-2299	ATCC	TEER	6×10^4^ cells/cm^2^
C8-D1A	CRL-2541	ATCC	TEER	Astrocyte Conditioned Media
Collagen from calf skin	C8919-20ML	Sigma/Millipore	TEER	1:4 in nanopure water
DMEM high glucose, pyruvate	11,995–073	ThermoScientific	TEER	500 mL
10% fetal bovine serum	10,082,139	Gibco	TEER	50 mL
L-glutamine	25,030,081	ThermoScientific	TEER	2 mM
Transwell-sterile	3,470	Costar Corning Inc.	TEER	24 well plates
xCT antibody-BSA Free	NB300-317	Novus	Immunoblotting	1:1000
α-tubulin antibody	3,873 s	Cell Signaling	Immunoblotting	1:5000
LI-COR IRDye 800CW Donkey anti-Rabbit	NC9523609	Fisher/LI-COR	Immunoblotting	1:10000
LI-COR IRDye 800CW Donkey anti-Mouse	NC0250903	Fisher/LI-COR	Immunoblotting	1:10000
MitoTracker^™^ Red CMXRo	M7512	Invitrogen	mitochondria visualization	final concentration of 250 nM in media
iScript cDNA Synthesis Kit	1,725,038	BioRad	qPCR	see manufacturer’s protocol
SsoAdvanced Universal SYBR Green Supermix	1,725,272	BioRad	qPCR	see manufacturer’s protocol
Phosphate Buffer Saline (PBS)	10,010,049	Gibco	General	General
ADRB2 forward primer	N/A	IDT	qPCR	5’-GTACCGTGCCACCCACAAGA-3’
ADRB2 reverse primer	N/A	IDT	qPCR	5’-CCCGGGAATAGACAAAGACCATC-3’
β-actin forward primer	N/A	IDT	qPCR	5′-GGCCAGGTCATCACTATTG-3′
β-actin reverse primer	N/A	IDT	qPCR	5′-GAGGTCTTTACGGATGTCAAC-3′

## 3 Methods

### 3.1 Animals

Studies were conducted with adult 8- to 20-week-old mice. Female naïve C57BL/6 J and B6.129P2-*Cnr2^tm1Dgen^*/J were purchased from The Jackson Laboratories. GFAP-Cre mice (*B6.Cg-Tg (Gfap-cre)77.6Mvs/2 J*) were also purchased from The Jackson Laboratories and bred with existing ADRB2^Flox/Flox^ mice (Cameron et al., 2019) to generate astrocyte-specific ADRB2 knockout mice on a C57BL/6 background. All animal care and experimental uses were approved and overseen by the Animal Care and Use Committee at University of Arizona Health Sciences (Tucson, A.Z., United States; IACUC). For astrocyte isolation, animals were euthanized using isoflurane followed by complete cervical dislocation in line with the standing IACUC protocol. For spinal cord injury, female 8–10-week-old C57BL/6 J mice were anesthetized with 10 mg/kg of ketamine and 6 mg/kg xylazine via i.p. injection. Vertebral columns were then exposed and stabilized, and a laminectomy of the 11th thoracic vertebrae was performed as previously described (Scholpa et al., 2019; Scholpa, 2023). A force controlled 60 kilodyne contusion with 0 s dwell time was performed using an Infinite Horizon IH-0400 impactor (Lexington, KY) with the dura intact (Scholpa, 2023). Astrocyte isolation was performed 72 h after injury.

### 3.2 Cell isolation

All information regarding materials including concentration, composition, and supplier information can be found in Tables 1–3.

Note: If plating cells for cell culture, pre-coat plates with collagen from calf skin (Table 3).

**Figure 1:**
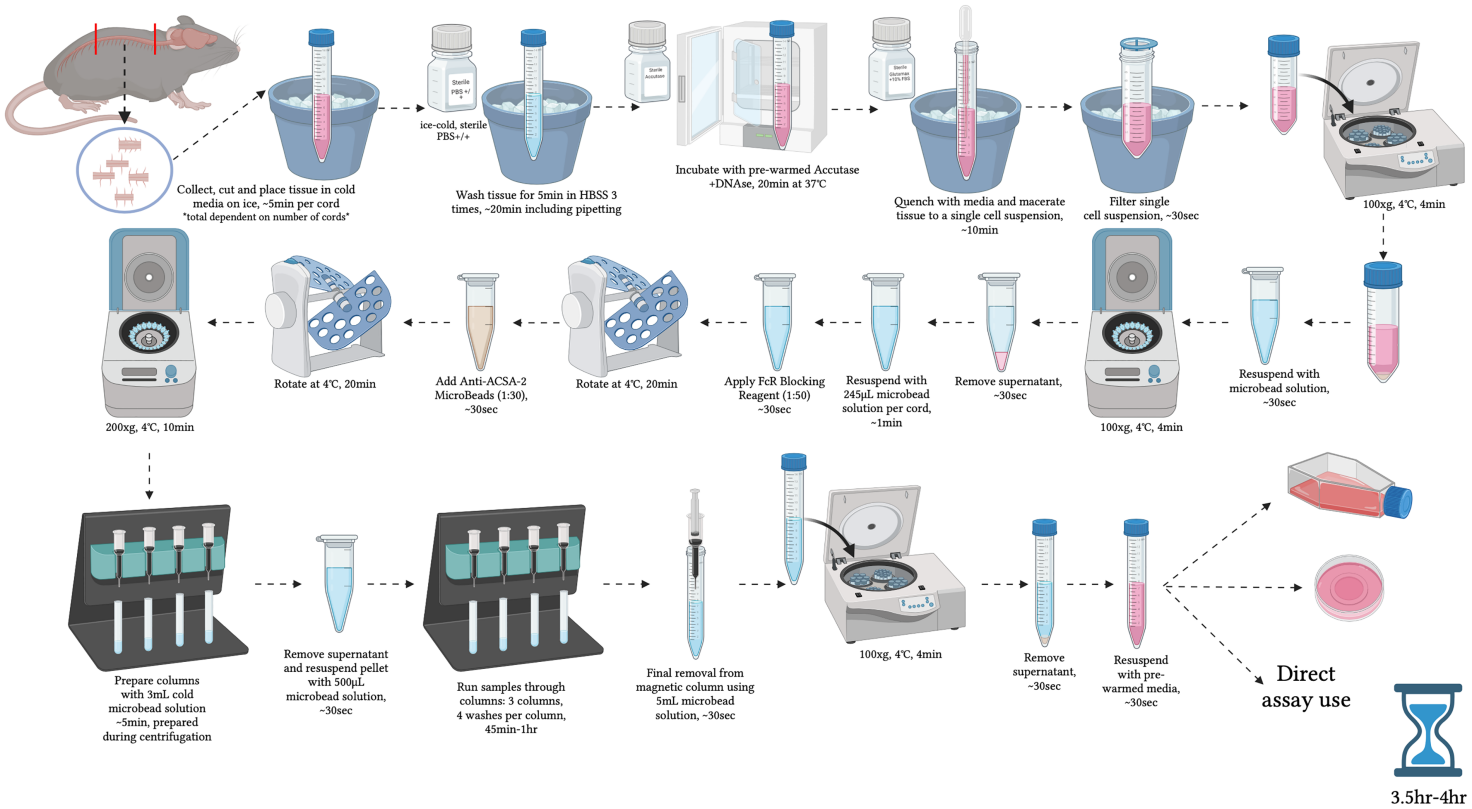
Graphical overview of the step-by-step procedure for primary adult mouse spinal cord astrocyte isolation. Created with BioRender.com.

Note: If plating cells for cell culture, remove collagen from plates and wash with sterile water. Then seed isolated cell suspension and centrifuge at 200 × g for 4 min at 4°C – for a more detailed description, refer to Section 3.3.

1. Prepare microbead solution as directed in the Miltenyi Biotec (Auburn, CA) Anti-ACSA-2 Microbead Kit for mice.
2. Filter magnetic microbead solution through 0.22 porous sterile filter in sterile hood and place in 4°C until needed.
3. Add 3 mL of DMEM/F12 GLUTAMAX with 10% FBS and 1% Pen/Strep, hereafter referred to as “complete media,” to one 15 mL falcon tube per cord and place on ice to chill.
  - This media was found to provide a higher yield over astrocyte-specific medias intended for immortalized cells or DMEM/F12 + 10% FBS alone.
4. Prepare the following reagents in the sterile hood per cord:
  - 8 mL DPBS +calcium/+magnesium (+/+) with 1% Pen/Strep on ice.
  - 2 mL accutase in sterile 37°C cell culture incubator to pre-warm.
  - 3 mL complete media in sterile 37°C cell culture incubator to pre-warm.
5. Collect spinal cord for isolation.
  - Isolations are performed in batches of no more than 8 cords at a time (Figure 1).
  - Euthanize mice as per section 3.1.
  - Remove entire spinal cord and cut into ~5 mm sections and place directly into chilled 15 mL falcon tube containing 3 mL complete media.
  - Harvesting recommendations:
    - After euthanasia, shave the animal’s back prior to first incision.
    - After exposing spinal column, use gauze to remove any loose hair and wipe down area with alcohol wipes before collection.
    - Vascular perfusion is not recommended and may reduce overall cell viability.
6. Move falcon tubes to sterile hood and remove media.
7. Wash cords using gentle agitation with pre-chilled DPBS+/+ with 1% Pen/Strep followed by slight manual rocking.
8. Leave to sit in DBPS +/+ on ice for 5 min.
9. Repeat steps 7 and 8 two additional times.
10. During final wash, prepare pre-warmed accutase+DNase.
  - 1:10 DNase to buffer into the accutase (Final DNase = [100unit/ml]).
11. After final wash, remove all DPBS+/+.
12. Add 2 mL of accutase+DNase per cord.
13. Place in sterile 37°C cell culture incubator for 20 min to enzymatically dissociate the tissue.
14. After incubation, neutralize the accutase with complete media (1,1) and place tubes on ice in sterile hood.
15. Triturate tissue gently 40–50 times using 3 mL transfer pipettes until single cell suspension is achieved.
  - Avoid generating bubbles as they can reduce cell yield.
  - Single cell suspension can be confirmed via microscopy by placing 10uL of sample onto a coverslip and viewing in brightfield.
  - Though myelin may still be present, trituration should be stopped once most of the sample has reached single cell suspension.
16. Place new 15 mL falcon tubes and 70-micron filters, one per cord, into sterile hood.
17. Filter triturated samples into the new tubes.
  - This should remove any clumps and myelin.
  - If combining two cords into one sample, filter and combine desired cords into one 15 mL falcon tube.
    - Whether or not to combine cords depends on desired yield and endpoint.
18. Centrifuge tubes at 100 × g for 4 min at 4°C.
19. Remove supernatant and resuspend cell pellet using 1.5 mL prepared magnetic microbead solution.
20. Centrifuge at 100 × g for 4 min at 4°C to wash pellet.
21. Remove supernatant and resuspend pellet with 245uL ice-cold magnetic microbead solution per cord.
  - If two cords were combined into one sample, resuspend with 490uL ice-cold microbead solution.
22. Place new cell suspension into sterile, labelled 2 mL microcentrifuge tubes.
  - If two cords were combined into one sample, separate the resuspended 490uL into two 2 mL tubes, 245uL per tube.
23. Block immune cells by adding FcR Blocking Reagent provided in the microbead kit at a 1:50 concentration.
24. Rotate tubes for 20 min at 4°C.
25. Add Anti-ACSA-2 MicroBeads at a 1:20 concentration.
26. Add 1.5 mL ice-cold magnetic microbead solution.
27. Centrifuge at 200 × g for 10 min at 4°C to wash cells, removing dead cells, debris, and un-bound microbeads.
28. During centrifugation, prepare the MACS Magnetic stand and magnetic LS columns.
  - Place a new 15 mL falcon tube under each column to catch flow-through.
  - Add 3 mL ice-cold microbead solution to each column and allow to elute out with gravity.
29. Once centrifugation is complete, remove and discard the supernatant.
30. Resuspend the cell pellets using 500uL ice-cold microbead solution.
  - If two cords were combined into one sample, resuspend each half of the sample separated in Step 22a with 250uL magnetic microbead solution, then combine the sample in one 2 mL microcentrifuge tube.
    - Resuspend each half of the separated sample.
31. Place each cell suspension into a column and allow to fully enter column.
32. Add 3 mL ice-cold magnetic microbead solution to the column and allow to elute out completely via gravity.
33. Repeat Step 33 three additional times using 3 mL of magnetic microbead solution each time to wash sample.
34. After the last wash, discard the falcon tube and prepare a new set of 15 mL falcon tubes.
35. Once the last wash has eluted out completely, remove the column from the magnetic stand and immediately flush out the column with 5 mL of magnetic microbead solution using the provided plunger into a new falcon tube – *this elution contains the cells.*
  - This needs to be done quickly but gently using medium pressure and speed.
  - Keep this flow-through.
36. Place newly prepared columns (see Step 29) in the magnetic stand with falcon tubes beneath.
37. Place the flow-through with cells collected in Step 36 into the new column and allow to elute out completely via gravity.
38. Repeat Steps 33–38 two additional times with a new column each time.
  - You will use three columns total per sample.
39. After the final elution, centrifuge flow-through sample tubes at 100 × g for 4 min at 4°C.
  - The pellet should be stable.
  - If performing more than four isolations, centrifuge in batches, keeping samples on ice.
40. Remove and discard supernatant.
41. Resuspend cell pellet in appropriate solution for desired endpoint, e.g.:
  - for plating: pre-warmed media to 37°C
  - for immediate RNA extraction: TRIzol solution
  - for protein isolation: preferred lysis buffer
  - etc.

Magnetic Microbead Solution Recipe (Final Concentration)**:

1. 1x PBS -calcium/−magnesium (−/−)
2. 0.5% BSA
3. 2 mM EDTA

**Filter solution through a 0.22 porous sterile filter and keep on ice or in 4°C. Solution can be stored at 4°C for up to one day**.

### 3.3 Cell culture

The average yield of live cells isolated from a single cord is approximately 825,000 cells. All cells maintained in culture were immediately plated on prepared collagen-coated 35 mm, 24-well or 96-well plates depending on desired outcomes. 35 mm plates were seeded with ~825,000 isolated cells, 24-well plates were seeded with 75,000 cells/well, and 96-well plates were seeded with 15,000 cells/well. Cell suspensions were seeded in the plate of choice and centrifuged at 200 × g for 4 min at 4°C to aid in cell adherence post-isolation. After 24 h a 50% media change was performed using pre-warmed complete media. For 35 mm plates, 50% media changes were performed every 48 h; in 24-well plates, every 24 h; and in 96-well plates, every 12 h.

### 3.4 Flow cytometry

Primary astrocyte purity was assessed by flow cytometry analysis, as previously described (Thompson et al., 2023). Briefly, proceeding primary isolation, cells were washed once with ice-cold 1× PBS−/− to remove any remaining microbead solution and pelleted at 100 × g for 4 min at 4°C. Supernatants were then aspirated, and cells were fixed/permeabilized in 1 mL of Image-IT^™^ Fixative Solutions containing 0.05% Triton^™^ X-100 for 10 min at room temperature (RT). Following fixation/permeabilization, cells were washed once with ice-cold 1× PBS−/− and pelleted at 100 × g for 4 min at 4°C. Supernatants were aspirated, cells were resuspended in 1 mL of ice-cold 1x PBS−/− supplemented with 1% FCS and 2 mM EDTA, and then counted utilizing a TC20 Automated Cell Counter (BioRad). Cells were then divided and strained into 5 mL Falcon round bottom polystyrene test tubes with a 35 μm nylon mesh cell strainer snap cap at a concentration of ~500,000 cells/tube. Cells were subsequently blocked with CD16/32 FcR-blocking antibody at a concentration of 1:50 for 20 min at 4°C, to mitigate non-specific binding. Next, cells were left either unstained or stained in one of the methods below.

For antibodies requiring one-step conjugation:

- Cells were stained with primary conjugated human anti-mouse ACSA-2-PE+ and GFAP-APC+ recombinant IgG1 monoclonal antibodies, or with recombinant human IgG1 PE+ and APC+ conjugated isotype control antibodies, at a dilution of 1:25 for 20 min rotating at 4°C, per manufacturer’s recommendation.
- Cells were stained with primary conjugated mouse anti-mouse IgG2aκ monoclonal antibody GLAST (ACSA-1)-PE+, or with mouse IgG2aκ PE+ conjugated isotype control antibody, at a dilution of 1:25 for 20 min rotating at 4°C, per manufacturer’s recommendation.

For antibodies requiring two-step conjugation:

- Cells were stained with rabbit anti-mouse IgG polyclonal antibody EAAT2 at a dilution of 1:50, recombinant rabbit anti-mouse monoclonal antibody IBA-1 at a dilution of 1:160, or rabbit anti-mouse IgG polyclonal antibody TMEM119 at a dilution of 1:500, for 1 h rotating at 4°C.
- Following primary conjugation, cells were washed with ice-cold 1× PBS−/− and pelleted at 100 × g for 4 min at 4°C to remove unbound primary antibodies.
- Cells were then counterstained with either Donkey anti-Rabbit IgG (H + L) Highly Cross-Adsorbed Secondary Antibody, Alexa Fluor^™^ Plus 488, Goat anti-Rabbit IgG (H + L) Highly Cross-Adsorbed Secondary Antibody, Alexa Fluor^™^ Plus 555, or Goat anti-Rabbit IgG (H + L) Cross-Adsorbed Secondary Antibody, Alexa Fluor^™^ 568 for 45 min rotating at 4°C.
- Unstained cells that underwent secondary counterstaining only were used as isotype controls for these markers.
- Following secondary conjugation, cells were then washed with ice-cold 1× PBS−/− and pelleted at 100 × g for 4 min at 4°C to remove unbound secondary antibodies.

After staining, cells were then washed twice with 1 mL of ice-cold FACS Solution to remove any remaining unbound antibodies and prepare cells for flow cytometry analysis. Cells were subsequently resuspended in 350 μL of FACS Solution. Primary cell isolations were then analyzed by flow cytometry analyses on a BD FACSCanto II flow cytometer to assess monoculture purity (see Table 2 for further solution and antibody details). Analyses and histograms were constructed by Flowjo V.10.9 software. Flow data was then cleaned in Flowjo via FlowAI/FlowClean plugins. All flow cytometry data was subsequently analyzed by employing standard gating from unstained controls across all test samples. Between 10,000–50,000 high quality single cell events were analyzed per sample/marker in Flowjo via the comparative histogram populations tool. Unstained and isotype controls were both utilized as comparators to ascertain the primary adult mouse spinal cord astrocyte purity. Purity is reported as percent positive cells utilizing the Overton cumulative histogram subtraction method (Overton, 1988). Chi-squared T (x) values ≥4 were considered statistical significant, as described by Flowjo (Roederer et al., 2001).

### 3.5 Immunocytochemistry

Immunocytochemistry was performed on cells cultured in 35 mm and 24-well plates. After 14 days in culture, media was removed, and cells were gently washed with 2 mL of pre-warmed, sterile DPBS+/+. Cells were then fixed and permeabilized using ImageIT Fixative solution containing 0.05% Triton x-100 for 10 min and then washed with RT DPBS three times for 5 min each time. After the final wash, ImageIT Fx-Signal-Enhancer was applied to the entire grow area and the plate was incubated in an RT, humidified chamber for 30 min. Plates were then washed once with 1 mL of 1x DPBS and cells were then blocked in 1 mL of pre-warmed BlockAid solution for 1 h at RT. BlockAid was removed and anti-EAAT2, anti-GFAP, anti-IBA-1 or anti-TMEM119 primary antibodies were prepared in fresh BlockAid and added to the fixed cultures at RT with gentle rocking for 1 h. Plates were then washed with RT DPBS three times for 15 min per wash. During the final wash, Goat anti-Rabbit IgG (H + L) Highly Cross-Adsorbed Secondary Antibody, Alexa Fluor^™^ Plus 647 (EAAT2), Donkey anti-Rabbit IgG (H + L) Highly Cross-Adsorbed Secondary Antibody, Alexa Fluor^™^ Plus 488 (GFAP), or Goat anti-Rabbit IgG (H + L) Highly Cross-Adsorbed Secondary Antibody, or Alexa Fluor^™^ Plus 555 (IBA-1, TMEM119) were diluted in fresh BlockAid solution. Secondary antibodies were subsequently added to their respective culture plates and allowed to incubate for 1 h at RT, protected from light, with gentle rocking. Plates were then washed with RT DPBS three times for 15 min per wash. Fluorescent conjugated primary antibodies anti-ACSA-2 and anti-ACSA-1 (GLAST) were diluted in fresh BlockAid solution, added to their corresponding culture plates, and allowed to incubate at RT for 30 min with gentle rocking. Plates were then washed with RT DPBS three times for 15 min per wash. Following antibody conjugation, cells were subsequently mounted with 400ul of Ibidi anti-fade mounting media with DAPI counterstain. Plates were then allowed to settle at 4°C overnight and imaged the following day on an EVOS-M5000 inverted microscope (Invitrogen).

### 3.6 Trans-epithelial electrical resistance functionality assessment

Conditioned media was collected from C8-D1A astrocytes (control), as well as astrocytes isolated from the spinal cords of naïve, SCI and CB2R KO mice. Brain endothelial cells (bEnd.3) were used at passages 9, 10, and 11 (*N* = 3). 24-well TEER plates with transwell inserts were prepared with collagen, washed with sterile water, and bEnd.3 s plated at 6×10^4^ cells/cm^2^ in DMEM with 10% FBS and 2 mM L-glutamine, and allowed to grow for 72 h. Astrocyte conditioned media from C8-D1A astrocytes, naïve astrocytes, SCI astrocytes, or CB2R KO astrocytes was subsequently added to the bottom section of the well for 24 h prior to recording TEER measurements. Measurements were recorded in triplicate daily for 4 days and the average value for each condition for each day reported.

### 3.7 xCT transporter assessment

Purchased C8-D1A and isolated naïve, SCI and CB2R KO astrocytes were collected 14d post-isolation/monoculture and then processed for immunoblotting as previously described (Slosky et al., 2016). The presence of xCT, a subdomain of the cysteine-glutamate antiporter (Slc7a11), was assessed using 4%–20% pre-made gradient gels at 150 V for 20 min followed by 190 volts for 30 min. Transfer was conducted using standing sandwich transfer in a cold environment at 20v for 65 min. The membrane was incubated with primary antibodies against xCT and α-tubulin with gentle rotation at 4°C, after which secondary antibodies were applied and incubated for 1 h at room temperature protected from light. Membranes were imaged using a Sapphire Biomolecular Imager (Azure Biosystems, Dublin, CA).

### 3.8 MitoTracker

After 14 days of monoculture, naïve astrocytes were stained with the live cell stain MitoTracker^™^ Red CMXRo per manufacturer’s protocol. Briefly, media was aspirated and replaced with basal media containing MitoTracker^™^ Red CMXRo at a final concentration of 250 nM. Cells were then placed in an incubator for 30 min at 37°C to facilitate dye integration. Following incubation, cells were washed three times with complete astrocyte media for 1 min per wash and immediately imaged on an EVOS-M5000 inverted microscope.

### 3.9 RNA expression

Total RNA was collected from isolated astrocytes using TRIzol regent per manufacturer’s protocol. Subsequently, cDNA was synthesized, and qPCR performed using the iScript cDNA Synthesis Kit and the SsoAdvanced Universal SYBR Green Supermix (BioRad), respectively. 500 ng of cDNA template was used across all samples and experiments. Primer sequences are denoted in Table 3.

## 4 Results

### 4.1 Flowcytometry

**Figure 2:**
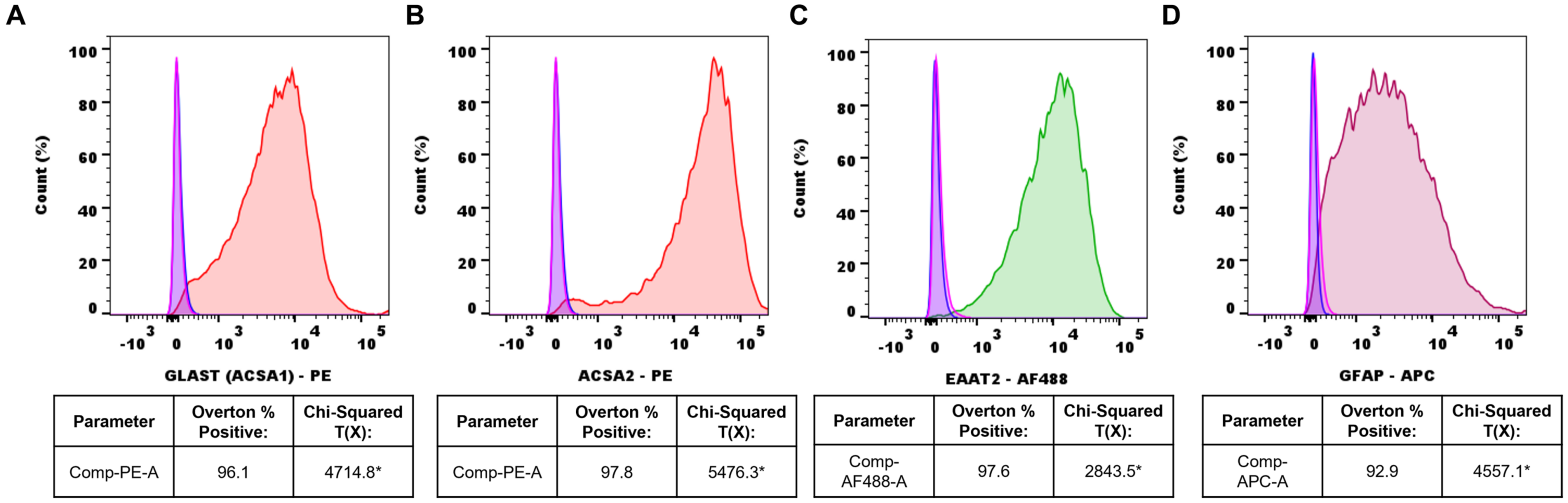
Representative flow cytometry histograms of isolated adult mouse spinal cord astrocytes. **(A)** ACSA-1-PE+ (red) astrocytes vs. IgG-PE+ isotype (pink) and unstained (blue) controls, **(B)** ACSA-2-PE+ (red) astrocytes vs. IgG-PE+ isotype (pink) and unstained (blue) controls, **(C)** EAAT2-AF488+ (green) astrocytes vs. IgG-AF488+ isotype (pink) and unstained (blue) controls, **(D)** GFAP-APC+ (burgundy) astrocytes vs. IgG-APC+ isotype (pink) and unstained (blue) controls. Data representative of *n*=3 samples/marker (*Chi-squared T(x) values ≥4 are considered statistically significant).

Following adult mouse spinal cord astrocyte isolation, cell purity was assessed via flow cytometry analyses. Isolated astrocytes displayed an average ACSA-1 purity of 96.1%, an ACSA-2 purity of 97.8%, an EAAT2 purity of 97.6%, and a GFAP purity of 92.9% compared to unstained and IgG isotype controls via the Overton cumulative histogram subtraction method (Figures 2A–D). In contrast, the statistical presence of known microglial markers TMEM119 and IBA-1 was not detected (Supplemental Figures S1A,B).

### 4.2 Astrocyte monoculture morphology

**Figure 3:**
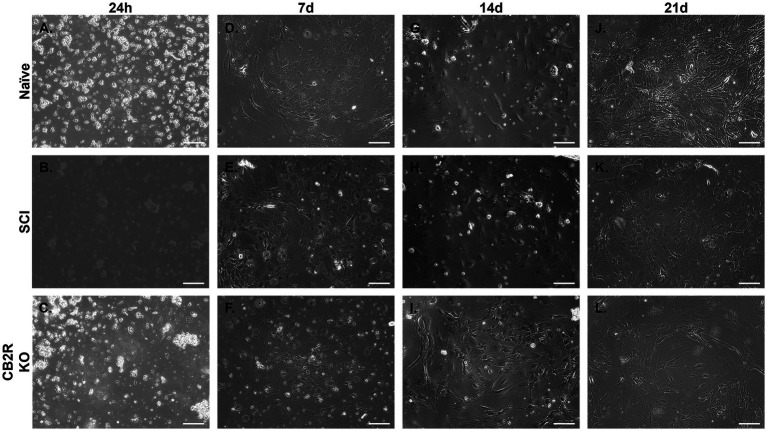
Representative phase-contrast photomicrographs of isolated adult mouse spinal cord astrocytes from various sources. Astrocytes grown in monoculture 24 h **(A–C)**, 7d **(D–F)**, 14d **(G–I)** and 21d **(J–L)**, depicting adhesion and cell growth over time. Images taken at 10x. Scale bar equal to 150 microns.

Spinal cord astrocytes isolated from naïve, SCI, and CB2R KO mice were harvested and immediately plated. Images were collected starting 24 h post-isolation (Figures 3A–C) and continued through 21 days. Naïve astrocytes were ~ 45% confluent 7d post-isolation (Figure 3D), ~85% confluent by day 14 (Figure 3G), and ~ 100% confluent by day 21 (Figure 3J). In the mice that had undergone SCI, the harvested astrocytes were ~ 40% confluent 7d post-isolation (Figure 3E), ~ 75% confluent by day 14 (Figure 3H) and ~ 95% confluent by day 21 (Figure 3K). Isolated astrocytes from CB2R KO mice grew at a slightly reduced rate. By day 7 they were 30% confluent (Figure 3F), by day 14 post-isolation ~65% confluent (Figure 3I), and by day 21, ~90% confluent (Figure 3L). While astrocytes are considered morphological and functional diverse (Lange et al., 2012), the cultures depicted above share key similarities in both outgrowth and morphology, suggesting that this isolation method produces viable and functional cells able to grow in morphologically appropriate ways.

### 4.3 Immunocytochemistry

**Figure 4:**
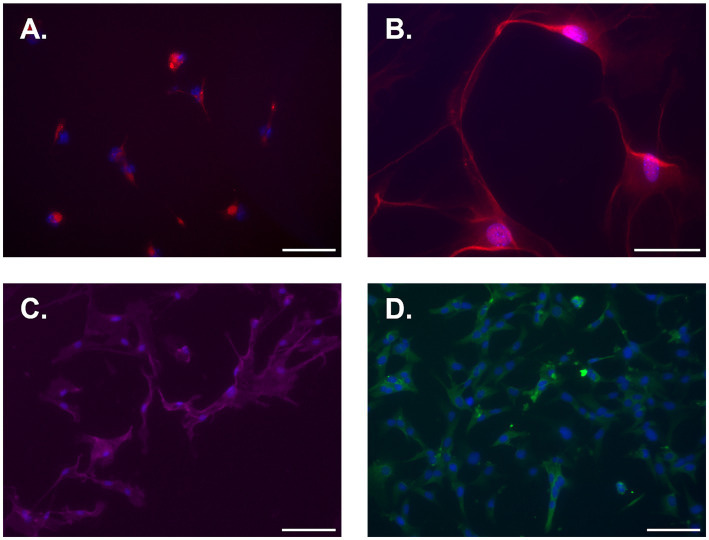
Representative immunofluorescent photomicrographs of naïve isolated adult mouse spinal cord astrocytes 7d post-isolation. **(A)** ACSA-1, **(B)** ACSA-2, **(C)** EAAT2, and **(D)** GFAP. Cells were counterstained with DAPI nuclear stain (blue). All images were taken at 20x except for ACSA-2, which was taken at 40x. Scale bar equal to 100 microns for **A, C**, and **D**, and 50 microns for **B**.

Female naïve spinal cord astrocytes were isolated, seeded, and cultured for 7d. Plates were then stained for astrocyte markers ACSA-1, ACSA-2, EEAT2, and GFAP. Immunofluorescent imaging of isolated astrocytes revealed high expression levels of all astrocyte markers examined (Figure 4). Conversely, no expression was observed for microglial markers TMEM119 and IBA-1 (Supplemental Figures S1C,D).

### 4.4 TEER functionality assessment

**Figure 5:**
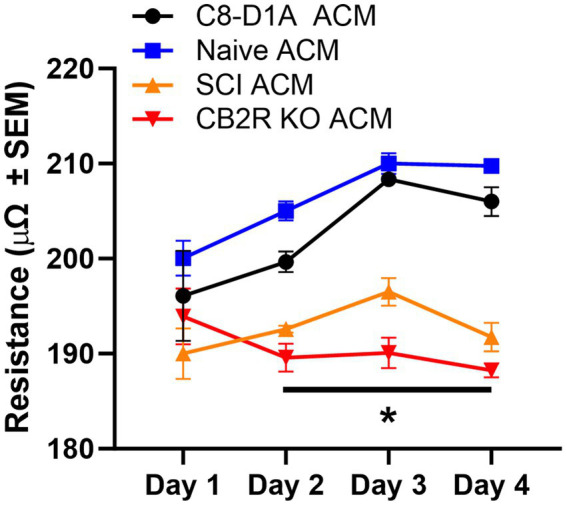
Trans-endothelial electrical resistance of bEnd.3 cells in the presence of astrocyte conditioned media. Astrocyte conditioned media (ACM) was collected from C8-D1A and isolated astrocytes from various sources (naïve, SCI, CB2R KO) and used for trans-endothelial electrical resistance (TEER) analysis of bEnd.3 cells. Data expressed as mean +/− SEM of *n* = 3 per group (*p ≤ 0.05 from C8-D1A and naïve isolation by Two-Way ANOVA followed by Tukey’s post-hoc test).

The blood-CNS barrier is a selectively permeable barrier that divides and protects the CNS from the outer environment (García-Salvador et al., 2020). This barrier maintains homeostasis and is made up of endothelial cells, astrocytes, pericytes, neurons, and microglial cells (García-Salvador et al., 2020). Astrocytes are considered vital to the maintenance and function of this barrier *in vivo*. Under *in vitro* conditions, endothelial cells in culture can lose their barrier properties, as indicated by reduced tight junction proteins and diminished trans-endothelial electrical resistance (TEER). Studies have shown that the loss of these properties can be rescued when cultured endothelial cells are treated with astrocyte conditioned media (ACM) or co-cultured with astrocytes (Heithoff et al., 2021). Therefore, TEER assays were conducted to ensure the ACM of isolated astrocytes can maintain endothelial cell properties similarly to ACM collected from purchased C8-D1A astrocytes. On Day 1, TEER was similar regardless of ACM source. This comparability was maintained between the purchased C8-D1A and the isolated naïve ACM on all days examined. On Days 2–4, however, SCI and CB2R KO ACM TEER was decreased (Figure 5). This was not unexpected, as the cells isolated from injured spinal cords are known to be functionally altered (Okada et al., 2018), and previous studies have shown that CB2Rs have modulatory roles in astrocytes, meaning the lack of this receptor could disrupt astrocyte ability to modulate endothelial cell properties (Cassano et al., 2017).

### 4.5 Protein expression of xCT

**Figure 6:**
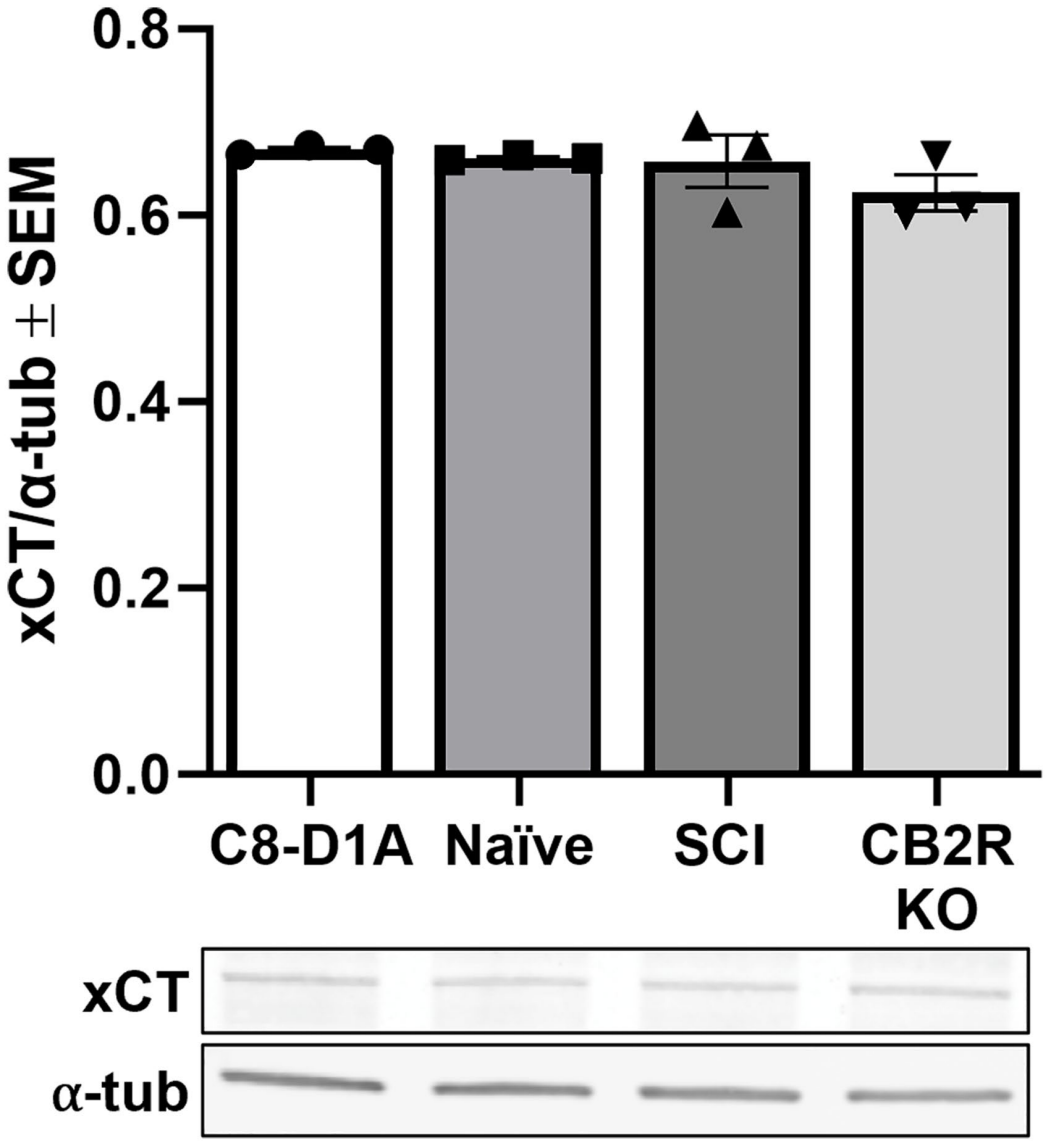
xCT transporter protein expression in purchased C8-D1A and isolated astrocytes. Astrocytes were isolated from various sources (naïve, SCI, CB2R KO) and collected for immunoblot analysis 14 days post-isolation. Data expressed as mean +/− SEM of *n* = 3 per group (*p* ≤ 0.05 by One-Way ANOVA followed by Tukey’s post-hoc test).

The cystine-glutamate antiporter (Slc7a11, xCT) is found throughout the CNS, predominantly in glial cells (Beckers et al., 2021), and is an important activator of excitatory neurotransmitter receptors, stimulating glutathione synthesis via catalyzation of cystine uptake and glutamate release (Ottestad-Hansen et al., 2018). The presence of xCT is considered a marker of functionality in *in vivo* and *in vitro* studies and dysregulation of this exchanger has been linked to a variety of neurological conditions (Beckers et al., 2021). Presence of xCT was confirmed in all cell sources tested and no significant differences were observed (Figure 6), further validating the retention of astrocyte functional markers when utilizing this isolation method, not only in naïve isolated cells but also SCI and CB2R KO cells.

### 4.6 MitoTracker and RNA expression studies

**Figure 7:**
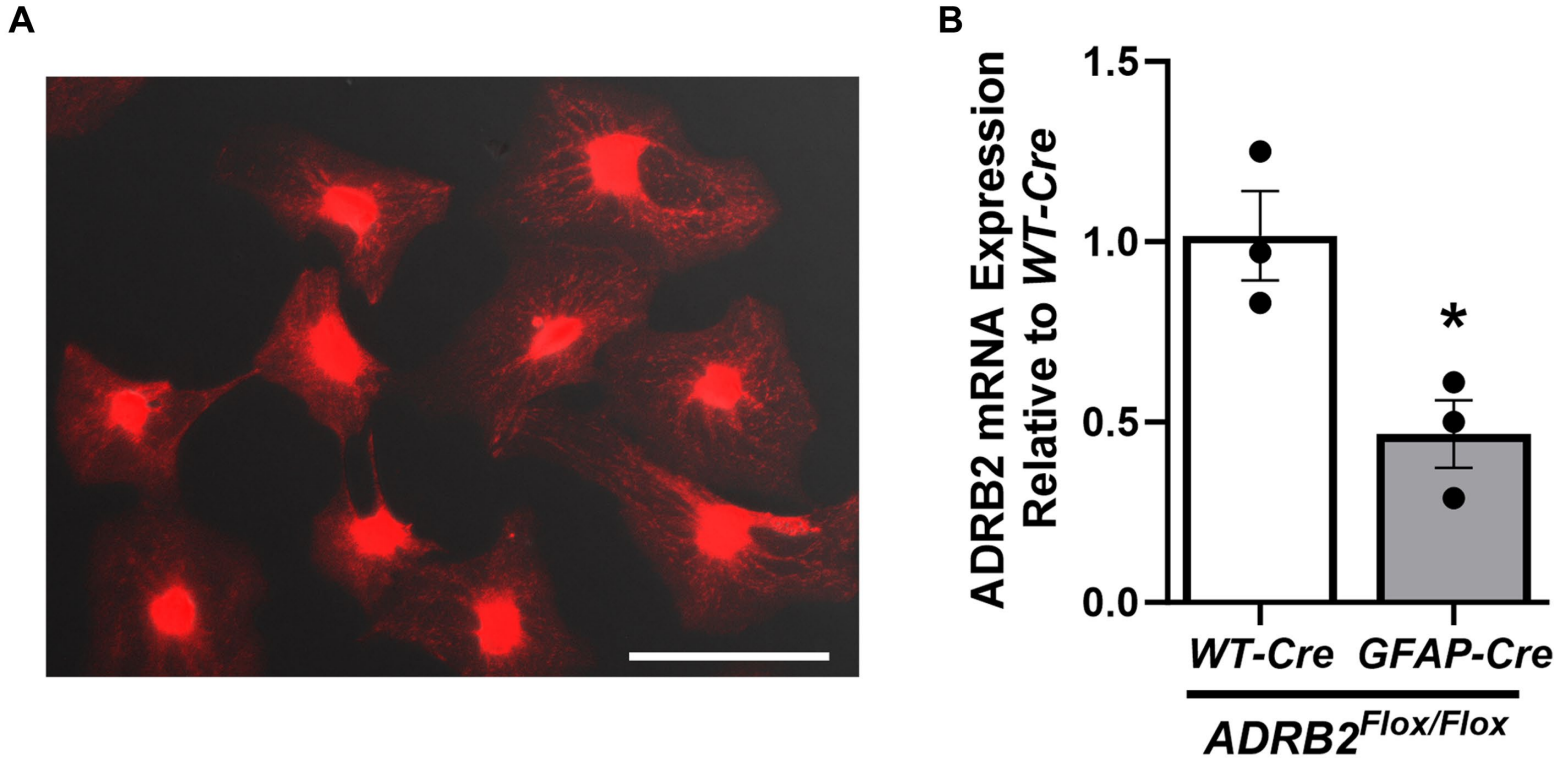
Presence of live mitochondria and β_2_-adrenergic receptor mRNA in isolated astrocytes. Active mitochondria were visualized in female naïve isolated adult mouse spinal cord astrocytes after 14d of culture **(A)**. mRNA expression of the β_2_-adrenergic receptor (ADRB2) was assessed in male astrocyte-specific ADRB2 knockout mice and littermate controls **(B)** via qPCR. Data expressed as mean +/− SEM of *n* = 3 per group (*p ≤ 0.05 by One-Way ANOVA followed by Tukey’s post-hoc test). Scale bar equal to 150 microns.

Astrocytic mitochondria play pivotal roles in oxidative phosphorylation, calcium (Ca^2+^) storage, and intracellular Ca^2+^ signaling and sequestration in astrocytes (Gollihue and Norris, 2020). Using MitoTracker^™^ Red CMXRos live cell staining, mitochondria were visualized in isolated naïve astrocytes 14d post-isolation (Figure 7A), indicating that the isolated astrocytes are both viable and mitochondrially active following prolonged monoculture.

Spinal cord astrocytes express the β_2_-adrenergic receptor (ADRB2), which is thought to enhance glycogenolysis and modulate intracellular energy transport (Jensen et al., 2016). Because ADRB2 plays a critical role in astrocyte function, ADRB2 mRNA expression was assessed via qPCR astrocytes isolated from male astrocyte-specific ADRB2 knockout mice (*GFAP-Cre/ADRB2^Flox/Flox^*) and littermate controls (*WT-Cre/ADRB2^Flox/Flox^*). As expected, ADRB2 expression was reduced in the astrocytes isolated from the knockout mice (Figure 7B).

## 5 Discussion

Challenges when isolating primary adult mouse spinal cord astrocytes include a reduction in yield with aging, smaller tissue volume of the spinal cord compared to the brain, and surrounding dense layers of myelin. Astrocyte cell surface antigen-2 (ACSA-2) is specifically expressed on mouse astrocytes and is considered a linage marker at all developmental stages (Kantzer et al., 2017). ACSA-2 has grown in popularity for studying and isolating neonatal and post-natal astrocytes from both the cortex and, more recently, the spinal cord (Batiuk et al., 2017; Ahn et al., 2022). Therefore, we developed an astrocyte isolation protocol that involves rapid tissue dissociation, minimal tissue manipulation prior to digestion to augment cellular yields, and magnetic-cell sorting via anti-ACSA-2 MicroBeads (Miltenyi Biotec) to improve isolation purity. The protocol presented within this manuscript allows for the isolation and monoculture of primary adult mouse spinal cord astrocytes that are suitable for various downstream histological, molecular, and functional applications.

Although primary isolation protocols for mouse spinal cord astrocytes have been published previously, there are limitations to these existing methods. For example, in contrast to our use of ACSA-2 MicroBeads, the protocol presented by Rosiewicz et al. (2020) relies on genetic modifications allowing for FACS based isolation, which alters the potential applicability. Additionally, their method resulted in a yield of approximately 10% of our observed yield. Furthermore, the authors utilized a more narrow age range (12–14 weeks), did not evaluate or report sex differences, and did not culture the isolated astrocytes (Rosiewicz et al., 2020). Ahn et al. (2022) recently reported a mouse spinal cord astrocyte isolation protocol utilizing ACSA-2 MicroBead sorting. Despite this similarity, that method required additional steps, including whole-body perfusion, removal of meninges, mincing of the spinal cord, density centrifugations, and a longer digestion time. The authors also specified that enzymatic dissociation must begin within 10 min of cardiac perfusion to prevent cell death, which is not a concern with our method. Additional differences with the aforementioned method include the use of dissociation kits with proprietary enzyme mixtures, fewer reported positive and negative astrocyte markers, and the lack of FcR blocker to mitigate non-specific cell labeling prior to magnetic purification (Ahn et al., 2022).

Unlike existing protocols, we performed astrocyte-specific downstream expression and functional assessments, and presented direct comparisons with available purchased astrocytes. Additionally, our technique relies on common cell-culture materials and does not require costly equipment or techniques, such as a perfusion pump, commercial dissociation kits, genetically modified reporter mice, or FACS sorting. We demonstrated the successful isolation of spinal cord astrocytes from both male and female mice, as well as in injured and genetically modified tissues, utilizing our novel spinal cord astrocyte isolation method. To model existing *in vivo* work, we utilized spinal cords from mice 8–20 weeks of age, with no observed impact on purity, viability, or yield due to aging. This versatility allows for a wide range of experimental applications, including co-culture studies, exploration of age-related alterations in disease models of choice, and studying both traumatic SCI and neurodegenerative disorders such as ALS or Huntington’s Disease, in which aggregation of astrocytes has been observed in the spinal cord (Gray, 2019). Importantly, the Miltenyi Biotec ACSA-2 Magnetic MicroBeads used in this protocol are marketed for and have been validated in brain tissue (Sharma et al., 2015; Holt and Olsen, 2016; Batiuk et al., 2017; Kantzer et al., 2017). Therefore, while not confirmed, the technique presented here could potentially be extended to mouse brain astrocyte isolation as well. Ultimately, this method could be used for a multitude of different models to achieve a broad-spectrum of desired experimental outcomes with the potential to aid in numerous basic and translational research endeavors.

## Data availability statement

The original contributions presented in the study are included in the article/Supplementary material, further inquiries can be directed to the corresponding author.

## Ethics statement

The animal study was approved by Animal Care and Use Committee at University of Arizona Health Sciences. The study was conducted in accordance with the local legislation and institutional requirements.

## Author contributions

IP: Formal analysis, Investigation, Methodology, Writing – original draft, Writing – review & editing, Conceptualization. AT: Conceptualization, Formal analysis, Investigation, Methodology, Writing – original draft, Writing – review & editing. NS: Formal analysis, Investigation, Methodology, Writing – original draft, Writing – review & editing. TL-M: Funding acquisition, Resources, Writing – original draft, Writing – review & editing. RS: Conceptualization, Funding acquisition, Project administration, Resources, Supervision, Writing – original draft, Writing – review & editing.

## Supplementary material

Supplemental Figure S1 Negative markers for isolated adult mouse spinal cord astrocytes. Representative flow cytometry histograms of **(A)** TMEM119-AF555+ astrocytes vs. IgG-AF555+ isotype (pink) and unstained (blue) controls and **(B)** IBA1-AF568+ astrocytes vs. IgG-AF568+ isotype (pink) and unstained (blue) controls. Representative IF-photomicrographs of **(C)** TMEM119 and **(D)** IBA-1.
