# Supplementary figures and images for: Isolation and monoculture of functional primary astrocytes from the adult mouse spinal cord
Source: Front Neurosci. 2024 Feb 16;18:1367473. doi: 10.3389/fnins.2024.1367473 (PMC10906264; doi:10.3389/fnins.2024.1367473)

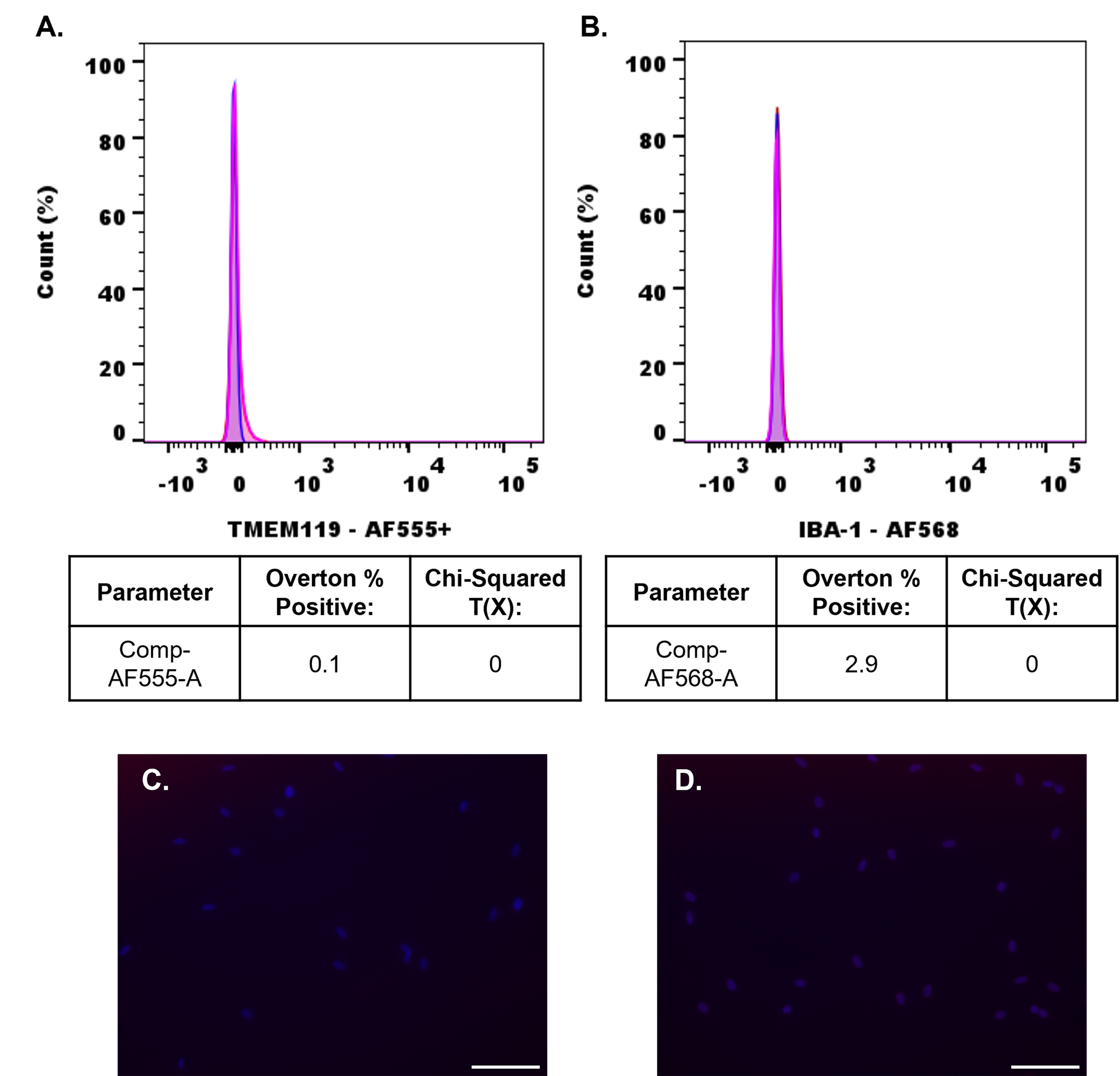

Supplement: Supplemental Figure S1 — Negative markers for isolated adult mouse spinal cord astrocytes. Representative flow cytometry histograms of (A) TMEM119-AF555+ astrocytes vs. IgG-AF555+ isotype (pink) and unstained (blue) controls and (B) IBA1-AF568+ astrocytes vs. IgG-AF568+ isotype (pink) and unstained (blue) controls. Representative IF-photomicrographs of (C) TMEM119 and (D) IBA-1. [file Image_1.TIF]
